# Detection of explosive picric acid *via* ESIPT-inhibited fluorescent chemosensor: theoretical insights, vapour phase detection and flexible indicator design

**DOI:** 10.1039/d5ra05689f

**Published:** 2025-11-05

**Authors:** Pavithra S, Keshav Semwal, Vishnu S, Raksha D. Salian, Partha Kumbhakar, Avijit Kumar Das

**Affiliations:** a Department of Chemistry, Christ University Hosur Road Bangalore Karnataka 560029 India avijitkumar.das@christuniversity.in; b Centre for Renewable Energy and Environmental Sustainability, Christ University Karnataka 560029 India; c Department of Physics and Electronics, Christ University Bangalore 560029 India

## Abstract

A fluorescent probe, (*E*)-2-((benzo[*d*]thiazol-2-ylimino)methyl)-5-(diethylamino)phenol (BMP), was designed and synthesized using 4-(diethylamino)-2-hydroxybenzaldehyde and benzothiazole-2-amine, and subsequently characterized for its selective turn-off response toward picric acid (PA). Upon the gradual addition of PA, significant changes in the absorption and fluorescence spectra were observed, marked by strong fluorescence quenching even in the presence of competing nitroaromatic compounds. BMP exhibited two absorption signals at 350 nm and 433 nm with a prominent emission band at 488 nm, attributed to excited-state intramolecular proton transfer (ESIPT), accompanied by a large Stokes shift of 138 nm. The interaction between PA and the hydroxyl group of BMP effectively suppressed the ESIPT process, leading to the observed spectral variations. The binding interactions were further confirmed through NMR spectroscopy and density functional theory (DFT) calculations. The ligand BMP has been utilized as a selective chemosensor for PA with a 2-fold reduction in fluorescence intensity and 19-fold increment in absorption intensity, showing a binding affinity of 2 × 10^4^ M^−1^ and strong quenching efficiency toward picric acid, with a Stern–Volmer constant (*K*_sv_) of 14.059 M^−1^ with a limit of detection (LOD) of 4.87 μM. For practical implementation, BMP was successfully employed in a dipstick-based detection format for vapor-phase sensing. Moreover, BMP-embedded polymer films demonstrated excellent potential as solid-state fluorescent sensors, exhibiting visible fluorescence quenching upon exposure to PA. Their rapid, time-dependent emission response under UV light allows for convenient, on-site detection using devices such as smartphones, making them highly promising for real-world applications in explosives detection and environmental monitoring.

## Abstract 2

A fluorescent probe, (*E*)-2-((benzo[*d*]thiazol-2-ylimino)methyl)-5-(diethylamino)phenol, was synthesized using 4-(diethylamino)-2-hydroxybenzaldehyde and benzothiazole-2-amine, and characterized for its selective turn-off response toward picric acid.

## Graphical abstract

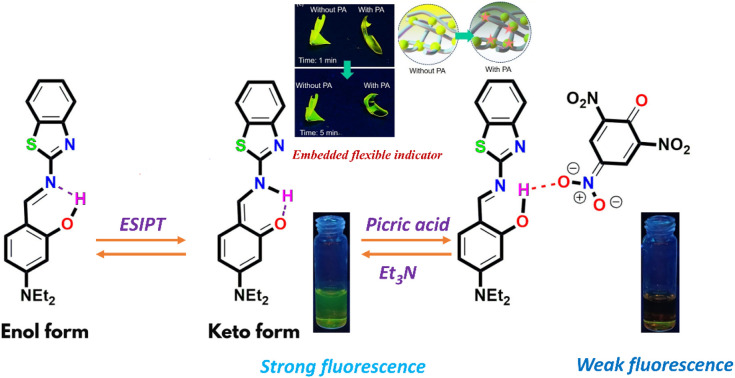

## 1 Introduction

**Scheme 1:**
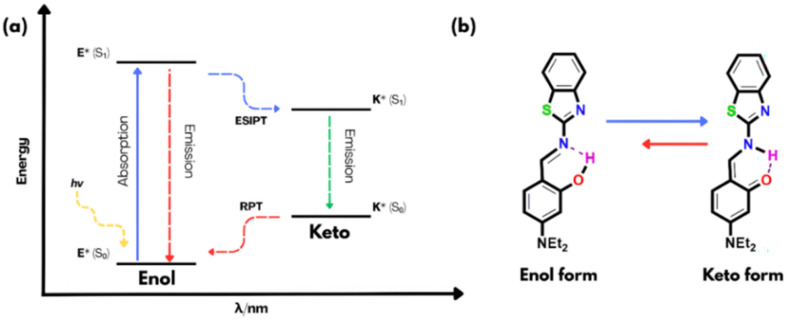
(a) Diagrammatic representation of the ESIPT process. (b) ESIPT phenomenon in the current molecule BMP.

2,4,6-Trinitrophenol or picric acid (PA) is a nitroaromatic compound that is a highly potent explosive, even more potent than TNT and is hence employed as a starting material for lethal weapons, rocket fuel and fireworks.^1^ Due to its high solubility in water, PA can easily seep into the groundwater and has been shown to be remarkably dangerous to all living beings and a high concentration of PA is carcinogenic and causes lung, skin and eye disorders.^2,3^ From the start of the 21st century, an increased effort has been made for the detection of nitroaromatic compounds to tackle terrorism, environmental pollution and removal of live land mines.^4^ Due to its highly electron-deficient nature, picric acid (PA) is resistant to degradation, and only limited efforts have been made to detect it selectively, as distinguishing PA from other interfering nitroaromatic compounds remains a significant challenge.^5,6^ Various spectroscopic^7^ and electrochemical methods have been employed for picric acid detection.^8^ Likewise, numerous sensing materials, including small molecules,^9^ nanoparticles,^10^ nanofibers,^11^ gels,^12^ polymers,^13^ and metal–organic frameworks (MOFs),^14^ have been developed for this purpose (Table S1). Among these, fluorescence-based sensors are particularly attractive due to their high sensitivity, low cost, portability, simple instrumentation, minimal sample preparation, and rapid response. As a result, this area has received significant research attention, leading to the development of many fluorescent sensors for picric acid over the past decade.^15,16^ These are molecular systems that give a considerable change in fluorescence on interacting with particular analytes exhibiting notable photophysical changes *via* various photophysical phenomena like PET, ICT, AIE, FRET and ESIPT, and new approaches are continually emerging in this field.^17,18^ In this context, excited-state intramolecular proton transfer (ESIPT) operates through intramolecular hydrogen bonding and involves a rapid (10^−15^ to 10^−12^ s) and reversible four-step photophysical process between the enol and keto forms (E → E*→ K*→ K)^19,20^ (Scheme 1).

ESIPT chromophores inherently exhibit a large Stokes shift and a short-lived emissive ground state (keto form). However, enhancing this Stokes shift through chemical modification remains a significant challenge.^21^ These characteristics minimize self-reabsorption and reduce inner filter effects, making it highly sensitive to its surroundings and overcoming the limitations of other detection methods.^22^ Common ESIPT fluorophores include derivatives of 2-(2′-hydroxyphenyl)benzimidazole, benzoxazoles, benzothiazoles, quinolines, benzophenones, flavones, anthraquinones, benzotriazoles, and others. However, although several reports exist on picric acid detection through fluorescence quenching mechanisms, to the best of our knowledge, only a few examples have been reported where picric acid is detected *via* inhibition of the ESIPT process.^23^ Therefore, we have designed and synthesized an ESIPT active fluorescent probe (*E*)-2-((benzo[*d*]thiazol-2-ylimino)methyl)-5-(diethylamino)phenol (BMP) using a simple Schiff base condensation reaction for selective detection of explosive PA by inhibition of ESIPT. NMR analysis has been performed to characterize the chemical structure of BMP (Fig. S2, SI).

## 2 Experimental

### 2.1 Materials and instrumentation

Highly pure reagents and solvents that are suitable for analytical and spectroscopic standards were procured from Sigma Aldrich and utilized directly without any additional purification. Melting point determinations was employed by means of a hot-plate apparatus accompanied by an open-ended capillary. ^1^H-NMR spectra were acquired *via* a Bruker instrument (400 MHz) using TMS as an internal reference compound and DMSO-d_6_ serving as the solvent. The coupling constants for ^1^H–^1^H are reported in Hertz (Hz), while the chemical shifts are expressed in delta units (*δ*). The fluorescence studies were carried out using a Shimadzu RF-5301 PC spectrofluorometer, while UV-vis titration experiments were conducted using a PerkinElmer Lambda 30 spectrophotometer. A standard 10 mm path length fluorescence cell was used during fluorescence analysis.

### 2.2 Experimental for BMP-embedded flexible indicator design

For making the BMP based flexible detector we have used BMP and TPU (thermoplastic polyurethane) polymer. The chemicals were purchased from Sigma Aldrich and used without further purification. For preparation of the BMP film: first, 0.1 g of BMP was dissolved in 2 mL of DMF solvent which was sonicated for 30 min. Simultaneously 3 mL of TPU solution was made using DMF solvent. For the preparation of the composite solution, 2 mL of BMP with 3 mL of TPU (2 : 3) ratio, was homogeneously mixed by stirring on a magnetic hot plate for 15 min at low temperature until it formed a gel-like suspension. The prepared suspension was than deposited on a glass substrate and dried at room temperature to get the BMP–TPU composite thin film.

### 2.3 Synthesis and characterization of BMP

**Scheme 2:**
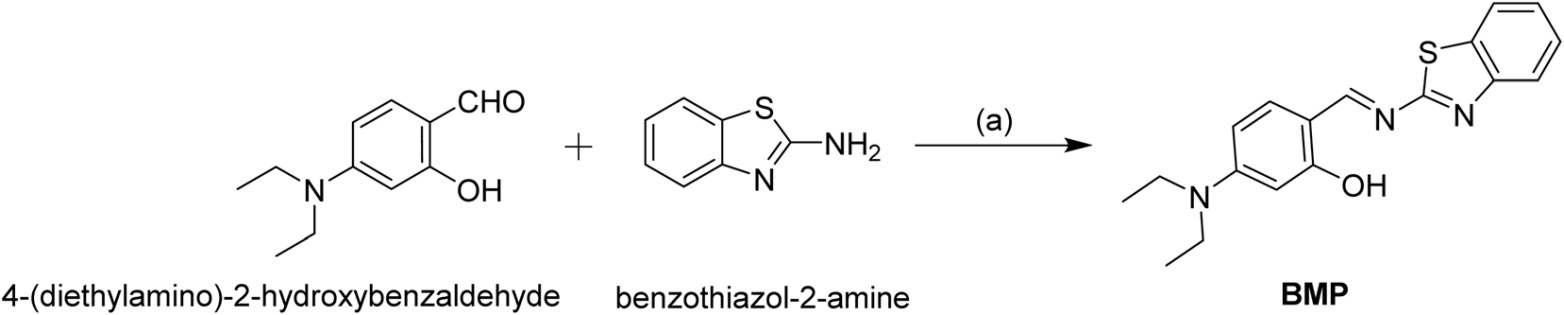
Synthesis of BMP: (a) MeOH, rt, 72 h.

To synthesize the probe BMP, 4-(diethylamino)-2-hydroxybenzaldehyde (0.1 g, 0.517 mmol) and benzothiazole-2-amine (0.077 g, 0.517 mmol) were reacted in methanol. Initially, 10 mL of methanol was used to dissolve 4-(diethylamino)-2-hydroxybenzaldehyde in a 100 mL round-bottom flask, followed by the addition of benzothiazole-2-amine. The reaction mixture was stirred at room temperature for 72 hours. Progress was monitored by thin-layer chromatography (TLC) on silica gel, with spot visualization under UV light. Upon completion, a precipitate formed, which was collected by filtration, washed with methanol, and dried under vacuum to yield a yellowish powder of BMP (Scheme 2). Yield: 150 mg, 90%, mp: 148 °C.

^1^H NMR (DMSO-*d*_6_, 400 MHz): *δ* (ppm): 12.39 (s, 1H, –OH), 9.07 (d, 1H), 7.99 (d, 1H, *J* = 8 Hz), 7.83 (d, 1H, *J* = 8 Hz), 7.59 (d, 1H, *J* = 9.2 Hz), 7.45 (t, 1H, *J* = 8 Hz), 7.35 (t, 1H, *J* = 8 Hz), 6.45 (dd, 1H, *J* = 12 Hz), 6.16 (d, 1H, *J* = 4 Hz), 3.46 (q, 4H, *J* = 8 Hz), 1.15 (t, 6H, *J* = 8 Hz).

## 3 Results and discussion

### 3.1 Solvatochromic analysis

In the solvatochromic analysis, the emission characteristics of the probe BMP were investigated using various solvents like acetone, hexane, methanol (MeOH), water, dimethyl sulfoxide (DMSO), acetonitrile (CH_3_CN), chloroform (CHCl_3_), ethyl acetate, ethanol (EtOH) and tetrahydrofuran (THF) to compare its fluorescence response in different solvent media. Significantly, variation of emission intensities and shifting of emission maxima have been recorded in various solvents. Among the tested solvents, BMP exhibited stronger fluorescence in ethyl acetate and ethanol as compared to other solvents at 495 nm (Fig. S9, SI). Although the emission of BMP showed minor shifts in different polar aprotic and protic solvents, no definite correlation was established between these spectral changes and specific solvent parameters. This finding suggests that various solvent properties, such as hydrogen bonding and polarity, contribute differently to the overall solvent effect.^24^

### 3.2 Spectroscopic response of probe BMP towards picric acid

**Fig. 1:**
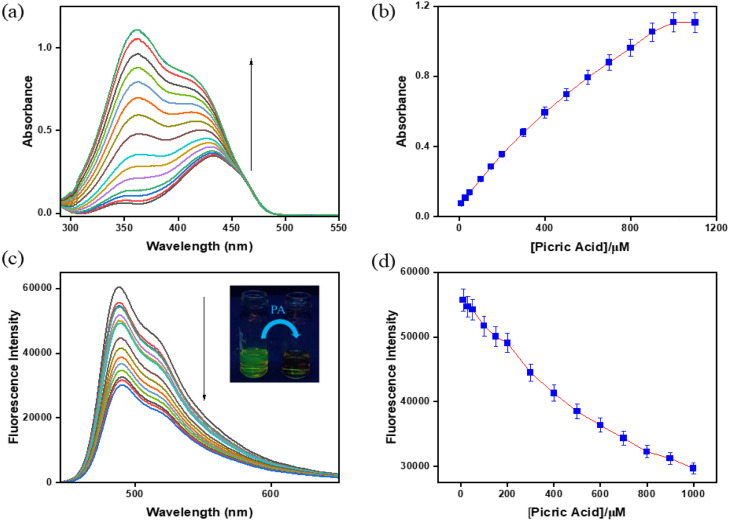
(a) UV-vis and (c) fluorescence spectra of BMP (*c* = 2.0 × 10^−5^ M) with picric acid (*c* = 2.0 × 10^−4^ M) in CH_3_CN/HEPES buffer solution (9 : 1, v/v, pH 7.4). Inset: the naked eye fluorescence change from green to colourless. (b) Variation in absorbance and (d) fluorescence with increasing concentration of picric acid (error value, 5%; *Y* error bar for both [±] deviation).

To demonstrate the interaction of BMP towards PA, UV-vis and fluorescence experiments have been performed. The absorption and emission properties of BMP (*c* = 2.0 × 10^−5^ M) in the presence of PA (*c* = 2.0 × 10^−4^ M) were investigated in a CH_3_CN/HEPES buffer mixture (9 : 1, v/v, pH 7.4), in which it exhibited a 2-fold reduction in the fluorescence intensity along with a 19-fold increment in absorption intensity. In the UV-vis spectral analysis, BMP initially showed a weak absorption peak at 350 nm and a strong peak at 433 nm. Upon gradual addition of picric acid, the absorption intensity at 350 nm increased significantly relative to that at 433 nm. This change in absorbance progressed with increasing PA concentration until a saturation point was reached (Fig. 1a and b).

In the fluorescence titration experiment, BMP exhibited a strong emission peak at 488 nm upon excitation at 433 nm, along with a relatively high fluorescence quantum yield (*Φ* = 0.33). With the incremental addition of PA, the emission intensity gradually decreased with a naked eye fluorescence change from green to colourless, indicating a quenching effect of approximately 2-fold with a low fluorescence quantum yield (*Φ* = 0.15). The stepwise addition of PA to the BMP solution resulted in a consistent reduction in fluorescence intensity at 488 nm, eventually reaching a saturation level (Fig. 1c and d).

According to linear regression analysis, the limit of detection (LOD) of BMP toward picric acid was determined to be 4.87 μM using the equation DL = *K* × Sb_1_/*S*, where *K* is taken as 3, Sb_1_ represents the standard deviation of the blank, and *S* is the slope of the calibration curve^25,26^ (Fig. S4, SI). The reaction rate constant between BMP and picric acid was calculated to be 514.83 s^−1^ based on the time-dependent fluorescence intensity changes upon stepwise addition of picric acid (Fig. S5, SI). Furthermore, Job’s plot confirmed a 1 : 1 binding stoichiometry between BMP and picric acid (Fig. S1, SI). The Benesi–Hildebrand plot was employed to determine the binding affinity constant, which was found to be 2 ×10^4^ M^−1^ (Fig. S6, SI).^27,28^ The probe demonstrated high quenching efficiencies, with Stern–Volmer constants (*K*_sv_) of 14.059 M^−1^ for PA (Fig. S7, SI).

### 3.3 Interference study

**Fig. 2:**
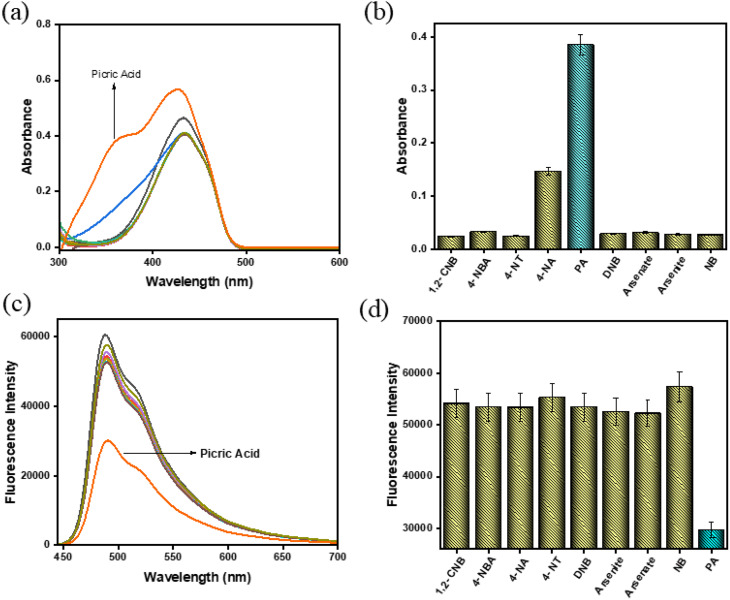
(a) UV-vis and (c) fluorescence spectra of BMP (*c* = 2.0 × 10^−5^ M) recorded after the addition of various potentially interfering analytes (*c* = 2.0 × 10^−4^ M, 15 equivalents) in CH_3_CN/HEPES buffer (9 : 1, v/v, pH 7.4). Corresponding (b) UV-vis and (d) fluorescence responses of BMP toward the different analytes are presented as bar diagrams (error value, 5%; *Y* error bar for both [±] deviation).

To evaluate the selectivity of BMP toward PA, an interference study was conducted using various potentially interfering analytes, including 1-chloro-2-nitrobenzene (1,2-CNB), 4-nitrobenzoic acid (4-NBA), 4-nitroaniline (4-NA), 4-nitrotoluene (4-NT), dinitrobenzene (DNB), arsenite, arsenate, and nitrobenzene (NB). These studies were carried out using UV-vis and fluorescence spectroscopy in CH_3_CN/HEPES buffer (9 : 1, v/v, pH 7.4). The BMP solution exhibited no significant absorption changes at 433 nm upon the addition of the various aromatic compounds. In contrast, only the addition of PA resulted in a strong absorption peak with a notable blue shift at 350 nm (Fig. 2a). The bar graph, based on UV-vis measurements, clearly illustrates the high selectivity of BMP for PA, as indicated by a prominent blue bar compared to the lower intensity yellow bars corresponding to other analytes. These results confirm that BMP exhibits strong and selective recognition of PA with minimal interference from structurally related compounds.

Similarly, in the fluorescence spectra, a significant quenching of emission at 488 nm was observed only upon the addition of picric acid, indicating strong interaction with BMP. In contrast, the addition of other interfering analytes caused no appreciable changes in fluorescence intensity (Fig. 2c). The bar diagram further highlights the pronounced selectivity of BMP toward PA: the lowest-intensity blue bar corresponds to the strong quenching response induced by PA, while the taller yellow bars represent the negligible responses from other analytes (Fig. 2d). These findings clearly demonstrate that BMP exhibits excellent selectivity and sensitivity toward picric acid, with minimal interference from structurally related compounds.

### 3.4 Competition experiment

**Fig. 3:**
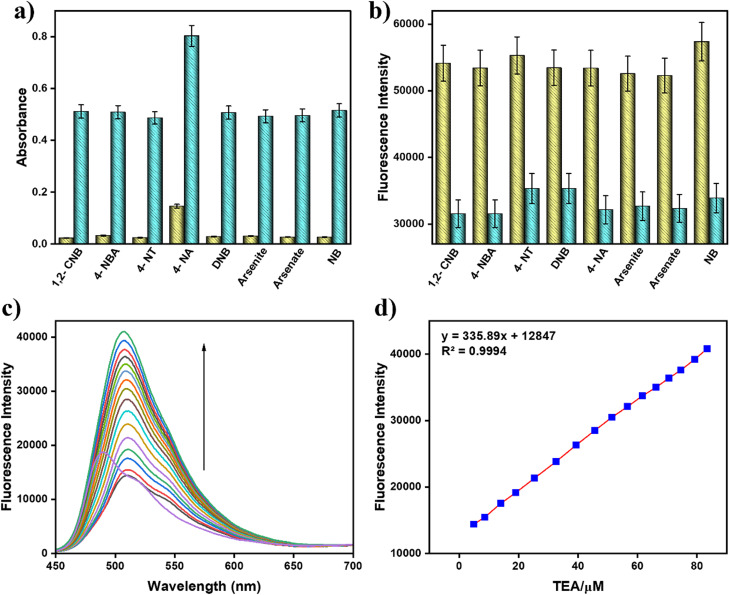
(a) Changes in absorbance of BMP (*c* = 2 × 10^−5^ M) + 15 equiv. of nitroaromatic compounds (yellow bars) (*c* = 2 × 10^−4^ M); changes in absorbance of BMP + 15 equiv. of nitroaromatic compounds and 10 equiv. of PA (green bars) (error value, 5%; *Y* error bar for both [±] deviation); (b) variation in fluorescence of BMP (*c* = 2 × 10^−5^ M) + 15 equiv. of nitroaromatic compounds (yellow bars) (*c* = 2 × 10^−4^ M); variation in fluorescence of BMP + 15 equiv. of nitroaromatic compounds and 10 equiv. of PA (green bars) (error value, 5%; *Y* error bar for both [±] deviation). (c) Fluorescence changes of BMP + PA (*c* = 2 × 10^−5^ M) on addition of triethylamine (*c* = 2 × 10^−4^ M). (d) Variation in emission intensity of BMP–PA solution with respect to triethylamine (TEA) concentration (error value, 5%; *Y* error bar for both [±] deviation).

To gain deeper insight into the selectivity of BMP towards picric acid (PA), UV-vis and fluorescence experiments were conducted in the presence of potentially interfering analytes using cross-contamination competition studies in CH_3_CN/HEPES buffer (9 : 1, v/v, pH 7.4). The selectivity of BMP for PA was assessed in the presence of various interfering species, including 1-chloro-2-nitrobenzene (1,2-CNB), 4-nitrobenzoic acid (4-NBA), 4-nitroaniline (4-NA), 4-nitrotoluene (4-NT), dinitrobenzene (DNB), arsenite, arsenate, and nitrobenzene (NB). Notably, the increase in absorbance and decrease in fluorescence intensity of BMP upon addition of PA remained largely unaffected by these competing analytes. In the bar diagrams, green and yellow bars represent the corresponding spectral responses of BMP to PA and other analytes respectively. These results demonstrate that BMP exhibits high selectivity and sensitivity toward PA, even in the presence of structurally similar or potentially interfering compounds (Fig. 3a and b).

To investigate the reversible nature of the interaction between BMP and picric acid (PA), fluorescence-based reversibility experiments were conducted. The study involved fluorescence titration using the BMP–PA complex and varying equivalents (0–2.0) of triethylamine added *in situ*. The results revealed that the strong fluorescence quenching observed in the BMP–PA complex was significantly reversed upon the addition of triethylamine. Due to the basicity of triethylamine, it forms a stable complex with PA and effectively displaces PA from the BMP binding site (Fig. 3c and d).^29^ Furthermore, this reversible behavior was maintained consistently even after four successive binding–release cycles, demonstrating the stability and robustness of the BMP system toward repetitive use (Fig. S10). These findings highlight the potential of BMP as an efficient and reusable fluorescent probe for picric acid detection.

### 3.5 Plausible binding mechanism of BMP with picric acid

**Scheme 3:**
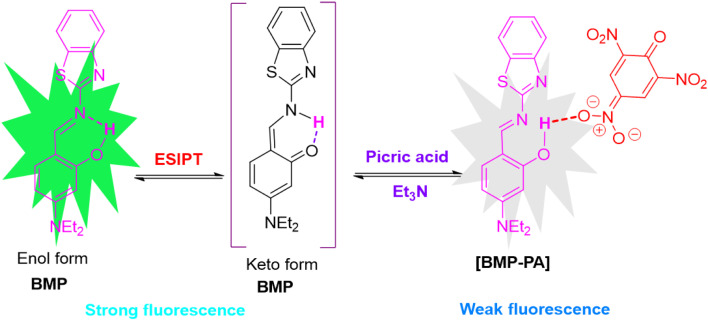
Probable binding mechanism of BMP with PA.

**Fig. 4:**
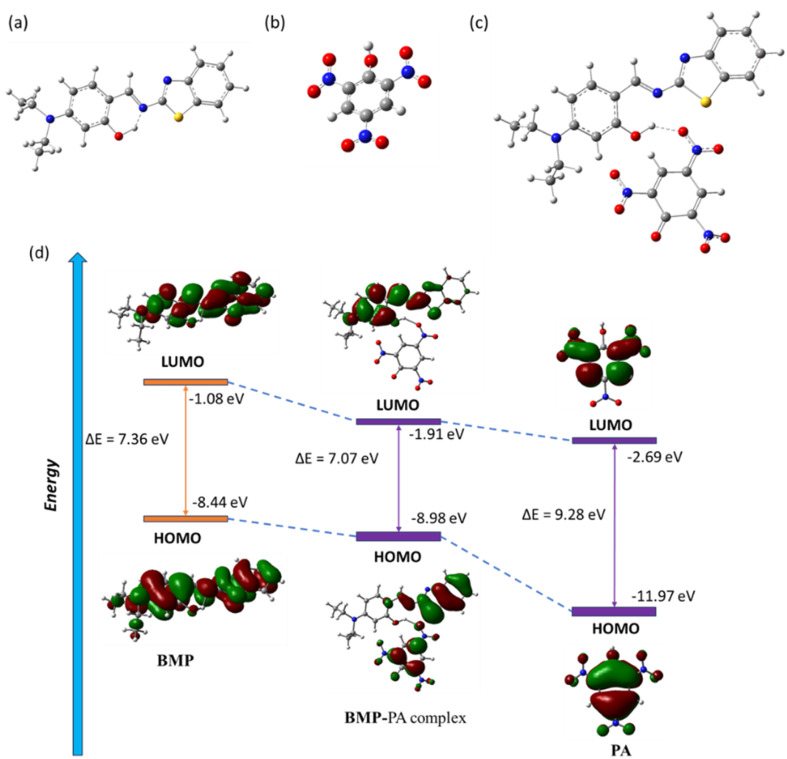
Geometry optimized molecular structures of (a) BMP, (b) PA and (c) BMP–PA complex. (d) Frontier molecular orbitals with energy differences of BMP, BMP–PA complex and PA.

To investigate the interaction between BMP and picric acid (PA), UV-vis, fluorescence, and ^1^H NMR studies were performed. In the absence of any analyte, BMP exhibited two absorption peaks at 350 nm and 433 nm, corresponding to the enol and keto forms, respectively, indicative of an excited-state intramolecular proton transfer (ESIPT) process. The emission maximum at 488 nm, with a large Stokes shift of approximately 138 nm, further supports the occurrence of the ESIPT phenomenon.^30^ Notably, the decrease in absorbance at 350 nm upon addition of picric acid suggests strong hydrogen bonding between the hydroxyl proton of BMP and the nitro groups of picric acid. BMP displays intense green fluorescence arising from an ultrafast photo-induced tautomerization *via* ESIPT, involving proton transfer from the hydroxyl group to the imine nitrogen through a six-membered transition state (Schemes 1 and 3). However, upon interaction with picric acid, significant changes in both the absorption and fluorescence spectra were observed—specifically, an increase in absorption intensity accompanied by pronounced fluorescence quenching from green to colorless. This quenching is attributed to hydrogen bonding interactions that suppress the ESIPT process (Scheme 3). These findings are further supported by theoretical calculations (Fig. 4). Additionally, ^1^H NMR analysis confirmed the strong interaction between BMP and PA. Upon addition of PA to BMP, the phenolic –OH proton signal nearly disappeared, indicating reduced electron density due to strong hydrogen bonding, while the aromatic proton signals remained unchanged (Fig. S3).

### 3.6 Theoretical study

To investigate the interaction mechanism between BMP and picric acid (PA), we conducted structural optimizations of both BMP and its complex with PA using Density Functional Theory (DFT) at the B3LYP/6-31G level, implemented in the Gaussian 09 W software package (see SI). The initial geometries were built based on previously optimized DFT structures of BMP and the BMP–PA complex (Fig. 4). The optimized structure of BMP displayed a significant HOMO–LUMO energy gap of 7.36 eV, with the HOMO at −8.44 eV and the LUMO at −1.08 eV. The optimized structure of PA showed a significant HOMO–LUMO energy gap of 9.28 eV, with the HOMO at −11.97 eV and the LUMO at −2.69 eV. Upon complexation with PA, the energy gap decreased to 7.07 eV (HOMO: −8.98 eV; LUMO: −1.91 eV), suggesting enhanced structural stabilization due to strong intermolecular hydrogen bonding interactions between the hydroxy group of BMP and nitro group of PA with a distance of 1.88 Å (Fig. 4). In the free BMP molecule, the frontier molecular orbitals (FMOs) were delocalized across the aryl system and the benzothiazole unit, indicating limited intramolecular charge transfer (ICT).

However, in the BMP–PA complex, a clear spatial separation of the FMOs was observed: the HOMOs were largely concentrated on the electron-deficient PA and the benzothiazole segment of BMP, while the LUMOs were mainly localized on the BMP moiety. This redistribution of orbital density highlights the strong interaction between BMP and PA, which perturbs the electronic structure asymmetrically and contributes to a distinct optical response upon PA binding.

### 3.7 Practical application

#### 3.7.1 Dipstick and vapour phase detection methods

To explore the practical applicability of BMP, indicator strips were prepared by immersing TLC plates in a BMP solution (*c* = 2 × 10^−5^ M). These strips exhibited intense fluorescence under UV light, which was significantly quenched upon exposure to picric acid (PA), demonstrating the ability of BMP to detect PA through fluorescence quenching (Fig. S8a). A key advantage of these indicator strips is their capacity to provide immediate qualitative results without requiring any instrumentation. In real-world scenarios, these strips offer a more convenient and rapid approach for explosives detection compared to the direct use of BMP in solution.

Vapour-phase detection was also employed to assess the sensitivity of BMP to PA in its gaseous form. A TLC strip coated with BMP was placed over the mouth of a glass vial containing solid PA and left undisturbed at room temperature for 24 hours, allowing the vapour to interact with the probe (Fig. S8b).^31^ A distinct quenching of fluorescence was observed across the entire surface of the strip (Fig. S8c). This uniform quenching indicates effective interaction between PA vapours and the BMP probe, leading to fluorescence suppression. These results highlight the potential of BMP for vapour-phase detection of nitroaromatic explosives such as picric acid.

Additionally, soils are the most common repository of unintentionally released chemical warfare agents and explosives from former battlegrounds.^32^ Hence, we performed real-time field soil analysis, to understand the selectivity for picric acid. 1 g of the field soil sample was placed in separate vials along with the ligand BMP (*c* = 2.0 × 10^−5^ M), in the presence of PA (*c* = 2.0 × 10^−4^ M). In the fluorescence study, as the concentration of picric acid increased, the corresponding emission intensity progressively decreased (Fig. S11a) and from this titration, the detection limit was calculated and found to be 9.57 μM (Fig. S11b). This clearly demonstrates that the probe BMP can be employed for real-time detection of picric acid in environmental matrices, particularly soil samples.

#### 3.7.2 BMP-embedded flexible indicator

**Fig. 5:**
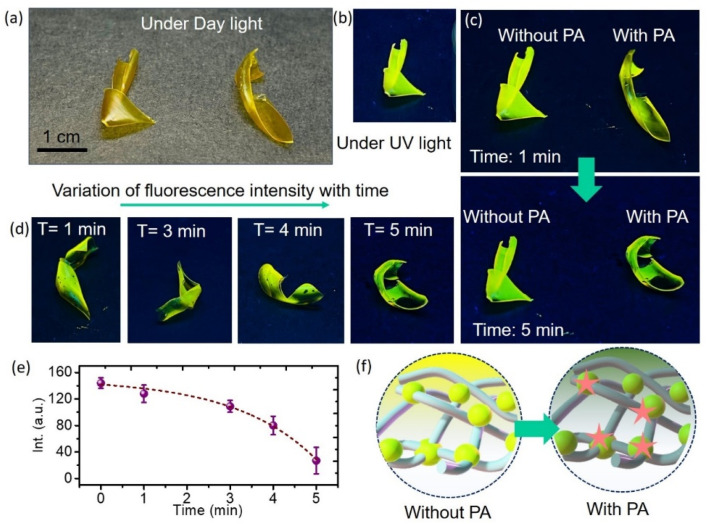
Digital images of the BMP–TPU film under (a) daylight and (b) 365 nm UV light irradiation. (c) Digital images of the film after addition of PA for 1 min. (d) Variation of fluorescence intensity with time. (e) Time dependent intensity variation in the presence of PA. (f) Schematic presentation of the interaction between PA and BMP molecules.

In the current work, the effects of PA on the visible luminescence of BMP in the presence of a solid polymer matrix have also been studied. This flexible polymeric membrane can act as an analytical indicator for rapid detection and real-time analysis of PA in hazardous conditions. We design the indicator using TPU polymer as a matrix embedded with BMP (for more details see the experimental section). The digital photographs of the yellow fluorescent BMP–TPU films under daylight and UV irradiation (365 nm) are displayed in Fig. 5a and b. For precise and sensitive analysis, solutions with concentrations of 20 μM of PA were added dropwise to the film. The intensity of the yellow color change of the film can be registered by a smartphone and the ImageJ software was used for signal quantification by measuring the amount of light intensity from the detection zone on the film surface. After adding PA for one minute, the resulting digital pictures are shown in Fig. 5c. The digital photos of the BMP–TPU film also exhibit the quenching of fluorescence in the presence of PA. It is evident from Fig. 5c, which depicts the change in the film’s emission intensity (digital pictures), that the polymer film’s intense yellow emission is rapidly quenched only in the presence of PA. In Fig. 5d, we have shown the changes in fluorescence intensity over time. After adding various concentrations of PA to solid flexible films for five minutes, the fluorescence intensity of the films under UV irradiation was measured and analysed. Fig. 5e displays a plot of the film’s time-dependent emission intensity, which shows a monotonic decrease in intensity over the time range under study. For further insight into the mechanism of fluorescence quenching, the diffusion coefficient was calculated for the BMP-embedded polymeric membrane using the time-lag method. The calculated values are ∼0.27 × 10^−5^ cm^2^ s^−1^ and 5.5 × 10^−5^ cm^2^ s^−1^ respectively for 1 min and 5 min. A higher diffusion value indicates a greater free space in the indicator. The strong luminescence of the film has been quenched drastically after the addition of the PA due to the strong interaction between the molecules and PA. Therefore, the time-dependent fluorescence change was provided by the interaction between PA and surface moieties of BMPs encapsulated inside the TPU matrix (Fig. 5f). The results indicate rapid sensing where analytes quickly reach the sensor’s active surface, improving the response rate, detection limit, and overall performance of the membrane.

## 4 Conclusion

In summary, the fluorescent probe BMP has been demonstrated as an efficient and selective sensor for the detection of picric acid (PA), exhibiting a low detection limit of 4.87 μM. The selective fluorescence quenching response arises from strong interactions between BMP and PA, which inhibit the ESIPT process, as supported by both spectroscopic and computational studies. The appearance of the two absorption signals with a significant blue shift from 433 nm to 350 nm and a large Stokes shift of 138 nm further confirm the operating ESIPT and the interaction mechanism. Practical applicability was showcased through dipstick and vapor-phase detection methods, enabling rapid, instrument-free identification of PA. Moreover, BMP-embedded TPU films serve as promising solid-state sensors, offering a visible and time-dependent fluorescence quenching under UV light, suitable for low-cost, on-site explosives detection using simple tools like a smartphone. Overall, the study highlights BMP as a versatile and robust probe with significant potential in environmental monitoring and field-deployable sensing technologies, contributing to the advancement of next-generation responsive materials.

## Conflicts of interest

There are no conflicts of interest to declare.

## Supplementary Material

RA-015-D5RA05689F-s001

## Data Availability

All data are available in the article itself and its supplementary information (SI). Supplementary information is available. See DOI: https://doi.org/10.1039/d5ra05689f.
