# Supplementary material for: Detection of explosive picric acid via ESIPT-inhibited fluorescent chemosensor: theoretical insights, vapour phase detection and flexible indicator design
Source: RSC Adv. 2025 Nov 5;15(50):42857–66. doi: 10.1039/d5ra05689f (PMC12587293; doi:10.1039/d5ra05689f)
Supplement: RA-015-D5RA05689F-s001 [file RA-015-D5RA05689F-s001.pdf]

Supplementary Information (SI) for RSC Advances.
This journal is © The Royal Society of Chemistry 2025

Detection of Explosive Picric Acid via ESIPT-Inhibited Fluorescent Chemosensor: Theoretical Insights, Vapour Phase Detection and Flexible Indicator Design

Pavithra S[a,b], Keshav Semwal[a,b], Vishnu S[a,b], Raksha D. Salian[c], Partha Kumbhakar[c], Avijit Kumar Das[a,b]*

[a] Department of Chemistry, Christ University, Hosur Road, Bangalore, Karnataka, 560029 India, Email: avijitkumar.das@christuniversity.in

[b] Centre for Renewable Energy and Environmental Sustainability, Christ University, Karnataka, 560029, India

[c] Department of Physics and Electronics, Christ University, Bangalore, 560029, India

Contents

01. Experimental **2**
1.1 General
1.2 General method of UV-vis and fluorescence titration
02. Determination of fluorescence quantum yield **2**
03. General procedure for drawing Job plot by fluorescence method **2-3**
04. ^{1}H NMR spectrum of BMP **3**
05. NMR titration study **4**
06. Calculation of the detection limit (DL), rate constant, inding constant and Stern-Volmer plot **4-6**
07. TLC plates photograph and vapor phase detection **6**
08. Solvatochromic analysis of BMP **7**
09. Reversibility cyclic studies **7**
10. Field soil Analysis **7**
11. Comparison table **8**
12. Computational details **9**
13. References **9-10**

1. **Experimental**

1.1. General method of UV-vis and fluorescence titration:

By UV-vis method:

For UV-vis titrations, stock solution of the sensor was prepared (*c* = 2 x 10^{-5} M) in CH_3CN-HEPES buffer (9/1, v/v, 25ºC) at pH 7.4. The solution of guest interfering analytes were prepared in the order of c = 2x10^{-4} M, including 1-chloro-2-nitrobenzene (1,2-CNB), 4-nitrobenzoic acid (4-NBA), 4-nitroaniline (4-NA), 4-nitrotoluene (4-NT), dinitrobenzene (DNB), arcenite, arcenate, nitrobenzene (NB) and picric acid (PA). Solutions of various concentrations containing sensor and increasing concentrations of nitroaromatics were prepared separately. The spectra of these solutions were recorded by means of UV-vis methods.

By fluorescence method:

For fluorescence titrations, stock solution of the sensor (*c* = 2 x 10^{-5} M) was prepared for the titration of nitroaromatic compounds (NACs) in CH_3CN-HEPES buffer (9/1, v/v, 25ºC) at pH 7.4. The solution of the guest analytes in the order of 2 × 10^{-4} M were also prepared. Solutions of various concentrations containing sensor and increasing concentrations of analytes were prepared separately. The spectra of these solutions were recorded by means of fluorescence methods.

2. **Determination of fluorescence quantum yield:**

Here, the quantum yield φ was measured by using the following equation,

$\varphi_x = \varphi_s (F_x / F_s)(A_s / A_x)(n_x^2/ n_s^2)$

Where,

X & S indicate the unknown and standard solution respectively,

φ = quantum yield,

F = area under the emission curve,

A = absorbance at the excitation wave length,

n = index of refraction of the solvent.

Here φ measurements were performed using anthracene in ethanol as standard [φ = 0.27] (error ~ 10%)

3. **General procedure for drawing Job plot by fluorescence method:**

For fluorescence titrations, stock solution of the sensor **BMP** (*c* = 2.0x10^{-5} M) was prepared for the titration of nitroaromatics in CH_3CN-HEPES buffer [9:1, v/v, pH = 7.4]. The solution of the guest analytes in the order of 200 μM were also prepared. Solutions of various concentrations containing sensor and increasing concentrations of nitroaromatics were prepared separately. The spectra of these solutions were recorded by means of fluorescence methods as shown in Fig.S1.

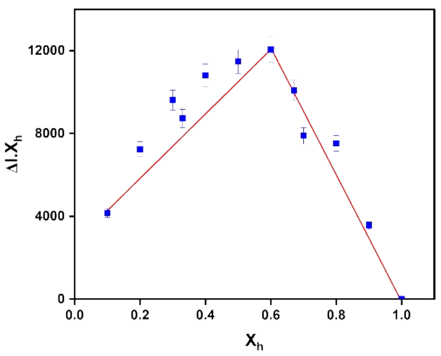

Figure S1. Job's plot diagram of receptor **BMP** for Picric acid (where Xh is the mole fraction of host **BMP** and ΔI indicates the change of the intensity) (error value, 5%; Y error bar for both [±] deviation).

4. ^{1}H NMR spectrum of BMP

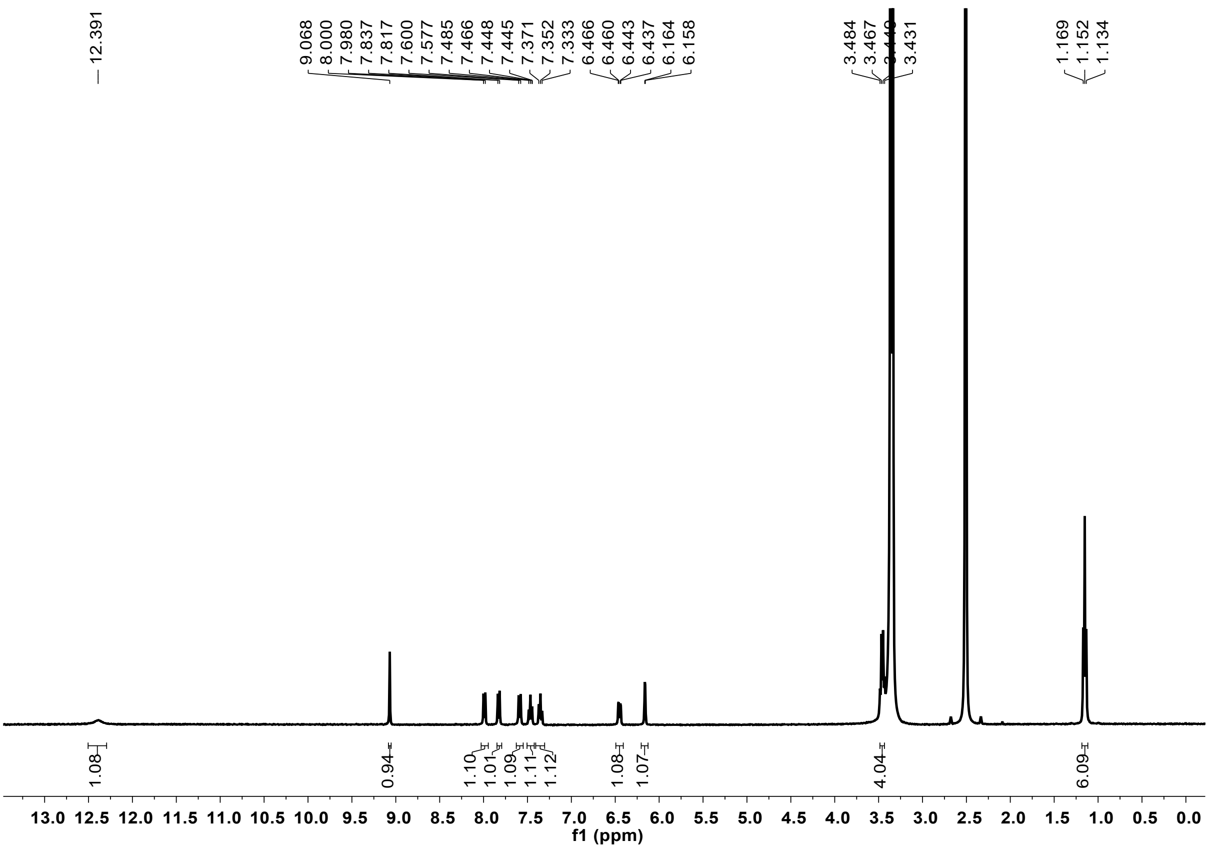

Figure S2. ^{1}H NMR spectrum of **BMP**

5. NMR titration study

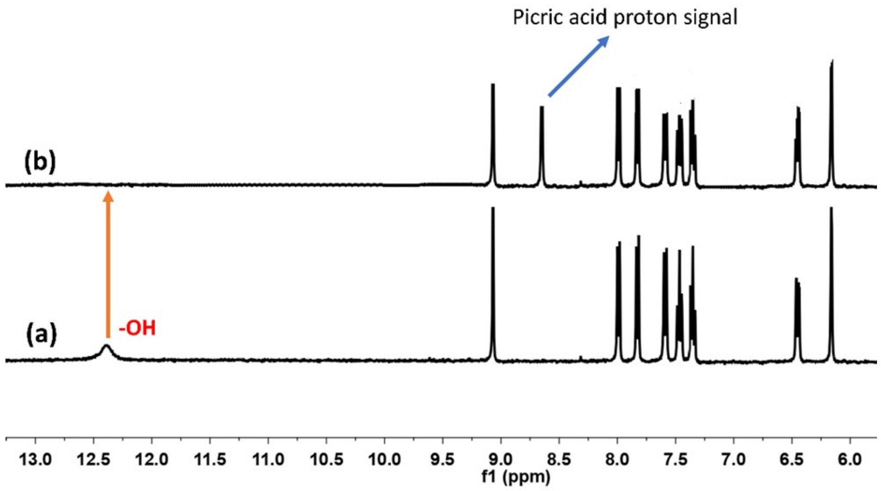

Figure S3. ^{1}H NMR study of **BMP** and **BMP** with PA in DMSO-d^{6}.

6. Calculation of the detection limit (DL):

The detection limit DL of **BMP** for picric acid was determined from the following equation:
DL = K* Sb1/S Equation-1
Where K = 2 or 3 (we take 3 in this case); Sb1 is the standard deviation of the blank solution; S is the slope of the calibration curve.
From the graph Fig.S4, we get slope = 406.34, and Sb1 value is 660.3281
Thus, using the formula we get the Detection Limit for Picric acid = 4.87 µM.

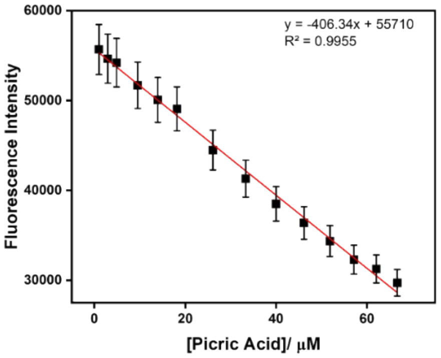

Figure S4. Changes of fluorescence intensity of **BMP** as a function of Picric acid (error value, 5%; Y error bar for both [±] deviation).

7. Rate constant calculation:

The changes of emission curve of **BMP** ($c = 2 \times 10^{-5}$M) at different time interval by the addition of PA and calculation of first order rate constant is displayed in Fig. S5. From the time vs. fluorescent intensity plot at fixed wavelength at 488 nm by using first order rate equation.

From Fig.S5 we get the rate constant for PA, K = slope × 2.303 Equation- 2

= 223.55 × 2.303 = 514.83 Sec^{-1}.

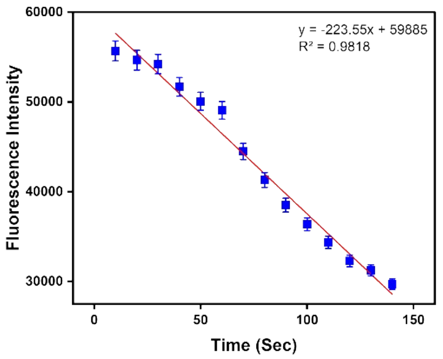

Figure S5. The first order rate equation by using time vs. fluorescent intensity of **BMP** with PA (error value, 2%; Y error bar for both [±] deviation).

8. Binding constant and Stern-Volmer plot

The binding constant value of Picric acid with the sensor has been determined from the emission intensity data following the modified Benesi–Hildebrand equation, $1/\Delta I = 1/\Delta I_{max} + (1/K[C])(1/\Delta I_{max})$ (Equation-3). Here $\Delta I = I - I_{min}$ and $\Delta I_{max} = I_{max} - I_{min}$, where I_{min}, I, and I_{max} are the emission intensities of sensor considered in the absence of guest, at an intermediate concentration and at a concentration of complete saturation of guest where K is the binding constant and [C] is the guest concentration respectively. From the plot of $(I_{max}-I_{min})/(I-I_{min})$ against $[C]^{-1}$ for sensor, the value of K has been determined from the slope. The association constant (K_a) as determined by fluorescence titration method for sensor with Picric acid is found to be 2×10^4 M^{-1} (error 5%).

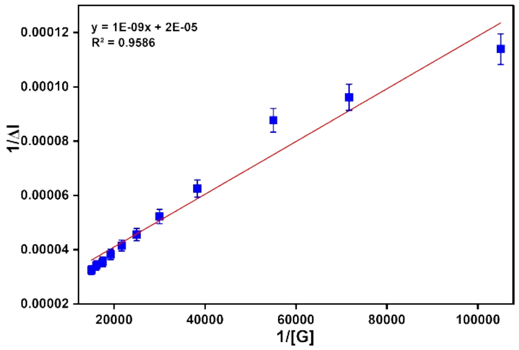

Figure S6. Benesi–Hildebrand plot from fluorescence titration data of receptor **BMP** (20μM) with picric acid (error value, 5%; Y error bar for both [±] deviation).

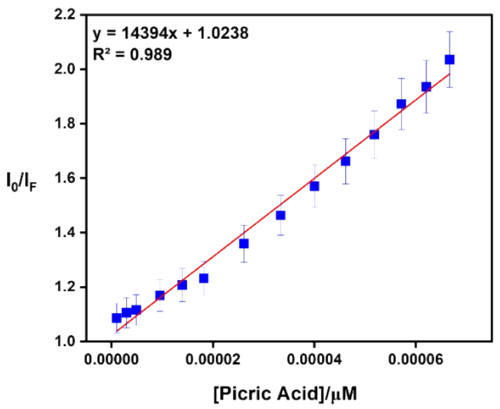

Figure S7. Stern-Volmer plot of **BMP** with picric acid (error value, 5%; Y error bar for both [±] deviation).

9. TLC plates photograph and vapor phase detection

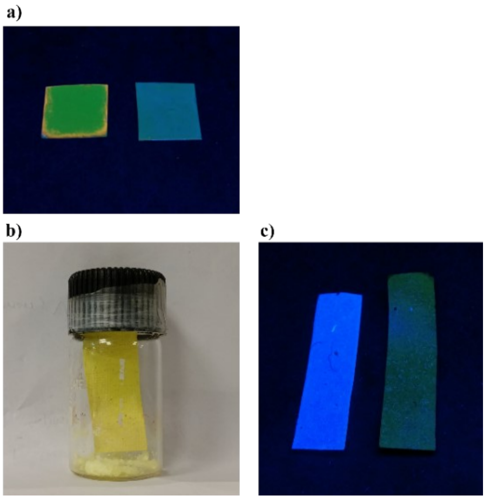

Figure S8. (a) TLC plates photograph with **BMP** ($c = 2.0 \times 10^{-5}$ M) at the left side and presence of picric acid ($c = 2.0 \times 10^{-4}$ M) at the right; (b) Picric acid vapor exposure inside a glass vial and (c) Fluorescence quenching observed in the presence of PA vapours (left: **BMP** coated TLC plate; right: **BMP** coated TLC plate + picric acid.

10. Solvatochromic analysis of BMP

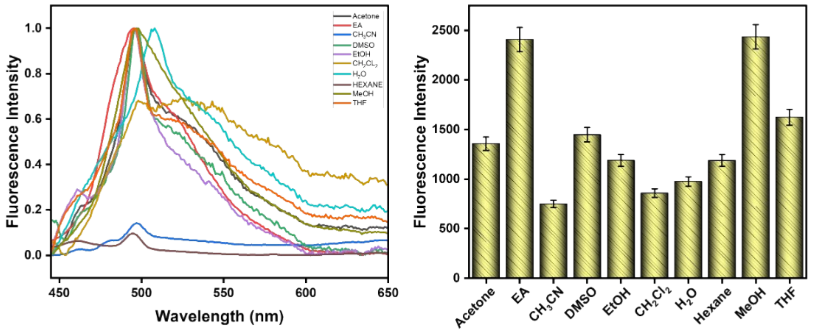

Figure S9. Solvatochromic analysis of **BMP** (error value, 5%; Y error bar for both [±] deviation).

11. Reversibility studies with various cycles

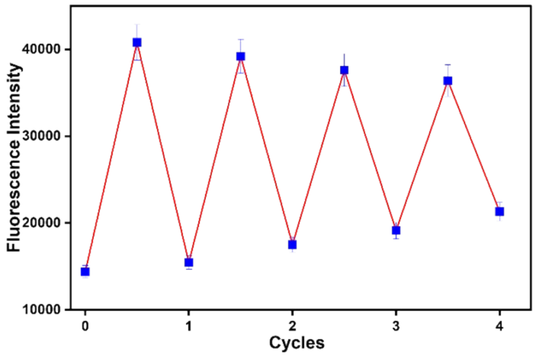

Figure S10. Changes of fluorescence intensity of **BMP** as a function of cycles (error value, 5%; Y error bar for both [±] deviation).

12. Field soil Analysis

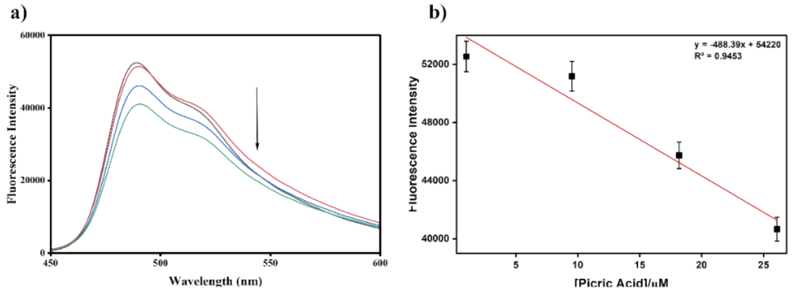

Figure S11. Fluorescence spectra of **BMP** (c = 2.0× 10^{-5} M) with field soil samples in CH_3CN/HEPES buffer solution (9:1, v/v, pH 7.4). (b) Changes of fluorescence intensity of **BMP** as a function of PA in field soil samples (error value, 2%; Y error bar for both [±] deviation).

13. Table S1: Comparison table of notable similarly reported sensors for detection picric acid

Molecular Structure	Fluorescence response	Colorimetric response	Range of wavelength	Application	Reference
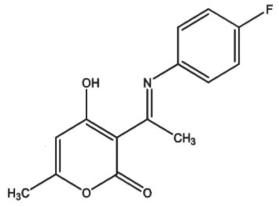	No fluorescence	UV-vis spectral changes	238 nm and 322 nm	Field application	6
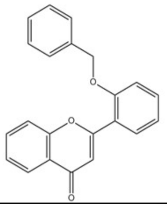	Turn off	Colourless to pale yellow	427-430 nm	Test Strips	7
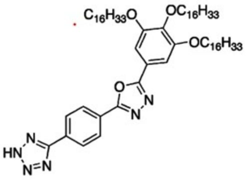	Fluorescence switching	Blue to green	414-486	Field application	8
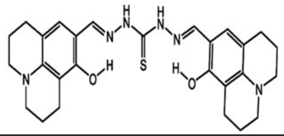	Ratiometric	Yellow to red	454 nm	Test strips	9
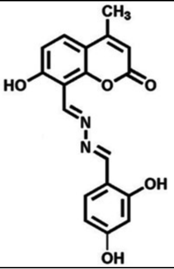	Turn off	-	429–493 nm	Water testing	10
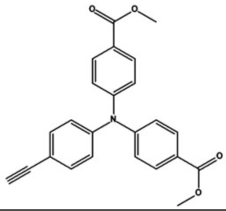	Turn-off	Colourless to yellow.	414-450 nm	Paper strip and vapour phase	11
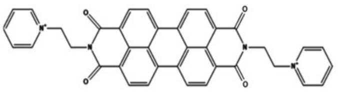	Turn off	Pink then fades and darkens	539 nm	Test strips	12
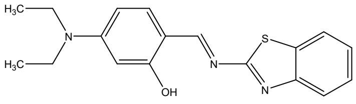	Turn off	Green to colourless	350 nm and 433 nm	Dipstick, vapour-phase and polymer based flexible indicator	This work

14. Computational details

Ground state electronic structure calculations in gas phase of the have been carried out using DFT[1] method associated with the conductor-like polarizable continuum model (CPCM).[2] Becke's hybrid function[3] with the Lee-Yang-Parr (LYP) correlation function[4] was used for the study. For H atoms we used 6-31+(g) basis set; for C, N, O atoms we employed LanL2DZ as basis set for all the calculations. The calculated electron-density plots for frontier molecular orbitals were prepared by using Gauss View 5.1 software. All the calculations were performed with the Gaussian 09W software package.[5]

15. References:

1. R. G. Parr, in *Horizons of Quantum Chemistry*, Springer Netherlands, Dordrecht, 1980, pp. 5–15.
2. (*a*) V. Barone and M. Cossi, *J. Phys. Chem. A.,* 1998, **102**, 1995; (*b*) M. Cossi and V. Barone, *J. Chem. Phys.*, 2001, **115**, 4708–4717; (*c*) M. Cossi, N. Rega, G. Scalmani and V. Barone, *J. Comput. Chem.*, 2003, **24**, 669–681
3. A. D. Becke, *J. Chem. Phys.*, 1993, **98**, 5648–5652.
4. C. Lee, W. Yang and R. G. Parr, *Phys. Rev. B Condens. Matter*, 1988, **37**, 785–789.
5. M. J. Frisch, G. W. Trucks, H. B. Schlegel, G. E. Scuseria, M. A. Robb, J. R. Cheeseman, G. Scalmani, V. Barone, B. Mennucci, G. A. Petersson, H. Nakatsuji, M. Caricato, X. Li, H. P. Hratchian, A. F. Izmaylov, J. Bloino, G. Zheng, J. L. Sonnenberg, M. Hada, M. Ehara, K. Toyota, R. Fukuda, J. Hasegawa, M. Ishida, T. Nakajima, Y. Honda, O. Kitao, H. Nakai, T. Vreven, J. A. Montgomery Jr., J. E. Peralta, F. Ogliaro, M. Bearpark, J. J. Heyd, E. Brothers, K. N. Kudin, V. N. Staroverov, R. Kobayashi, J. Normand, K. Raghavachari, A. Rendell, J. C. Burant, S. S. Iyengar, J. Tomasi, M. Cossi, N. Rega, J. M. Millam, M. Klene, J. E. Knox, J. B. Cross, V. Bakken, C. Adamo, J. Jaramillo, R. Gomperts, R. E. Stratmann, O. Yazyev, A. J. Austin, R. Cammi, C. Pomelli, J. W. Ochterski, R. L. Martin, K. Morokuma, V. G. Zakrzewski, G. A. Voth, P. Salvador, J. J. Dannenberg, S. Dapprich, A. D. Daniels, Ö. Farkas, J. B. Foresman, J. V Ortiz, J. Cioslowski and D. J. Fox, Gaussian Inc., 2009, Wallingford CT.
6. A. Goel and R. Malhotra, J. Mol. Struct., 2022, 1249, 131619.
7. V. Gopal, J. Sudhakaran, N. Ramachandran, T. K. Mana, A. R. Kana, A. O. Nair, P. Mohan,

T. Madhusudhan, S. Shanmugaraju and P. Nanjan, Sens. Diagn., 2024, 3, 1263–1271.

8. S. Nath, S. K. Pathak, B. Pradhan, R. K. Gupta, K. A. Reddy, G. Krishnamoorthy and A. S. Achalkumar, New J Chem, 2018, 42, 5382–5394.

9. K. Maiti, A. K. Mahapatra, A. Gangopadhyay, R. Maji, S. Mondal, S. S. Ali, S. Das, R. Sarkar, P. Datta and D. Mandal, ACS Omega, 2017, 2, 1583–1593.
10. P. P. Soufeena, T. A. Nibila and K. K. Aravindakshan, Spectrochim. Acta A Mol. Biomol. Spectrosc., 2019, 223, 117201
11. A. Chowdhury and P. S. Mukherjee, J. Org. Chem., 2015, 80, 4064–4075.
12. W. Zheng, Y. Zhao, X. Lin, Q. Zhang, K. Xiao, N. Cheng, X. Mei, Y. Lu and Z. Yao, Dyes Pigm., 2022, 207, 110761.
